# Association Between Extracellular Superoxide Dismutase Activity and 1-Year All-Cause Mortality in Patients With Acute Exacerbations of Chronic Obstructive Pulmonary Disease: A Prospective Cohort Study

**DOI:** 10.3389/fmed.2022.811975

**Published:** 2022-03-14

**Authors:** Haiqing Li, Wei Hong, Zixiong Zeng, Shan Gong, Fan Wu, Zihui Wang, Heshen Tian, Juan Cheng, Ruiting Sun, Mi Gao, Chunxiao Liang, Weitao Cao, Guoping Hu, Yuqun Li, Liping Wei, Yumin Zhou, Pixin Ran

**Affiliations:** ^1^Department of Respiratory Medicine, The Third Affiliated Hospital of Guangzhou Medical University, Guangzhou, China; ^2^State Key Laboratory of Respiratory Disease, National Center for Respiratory Medicine, National Clinical Research Center for Respiratory Disease, Guangzhou Institute of Respiratory Health, The First Affiliated Hospital of Guangzhou Medical University, Guangzhou, China; ^3^GMU-GIBH Joint School of Life Sciences, Guangzhou Medical University, Guangzhou, China

**Keywords:** AECOPD, extracellular superoxide dismutase (ecSOD), *SOD3*, mortality, prognosis

## Abstract

**Background and Objectives:**

Accumulating evidence suggests that oxidative stress is involved in the development of chronic obstructive pulmonary disease (COPD) and its progression. Activity of extracellular superoxide dismutase (ecSOD), the only extracellular enzyme eliminating superoxide radicals, has been reported to decline in acute exacerbations of COPD (AECOPD). However, the association between serum ecSOD activity and 1-year all-cause mortality in AECOPD patients remains unclear. The objective of our study was to explore the usefulness of ecSOD activity on admission in AECOPD as an objective predictor for 1-year all-cause mortality.

**Methods:**

We measured serum ecSOD activity in AECOPD patients on admission in a prospective cohort study. We also recorded their laboratory and clinical data. Multivariate Cox regression was used to analyze the association between ecSOD activity and the risk of 1-year all-cause mortality. Restricted cubic spline curves were used to visualize the relationship between ecSOD activity and the hazard ratio of 1-year all-cause mortality.

**Results:**

A total of 367 patients were followed up for 1 year, and 29 patients died during a 1-year follow-up period. Compared with survivors, the non-survivors were older (79.52 ± 8.39 vs. 74.38 ± 9.34 years old, *p* = 0.004) and had increased levels of tobacco consumption (47.07 ± 41.67 vs. 33.83 ± 31.79 pack-years, *p* = 0.037). Having an ecSOD activity ≤ 98.8 U/ml was an independent risk factor of 1-year all-cause mortality after adjustment for baseline differences, clinical variables and comorbidities [hazard ratio = 5.51, 95% confidence interval (CI): 2.35–12.95, *p* < 0.001].

**Conclusion:**

Lower serum ecSOD activity was a strong and independent predictor of 1-year all-cause mortality in AECOPD patients.

## Introduction

Chronic obstructive pulmonary disease (COPD), a systemic inflammatory disease, is the third leading cause of mortality worldwide (1). Exacerbations of COPD accelerate the decline in lung function and cause progression of the disease, reduced physical activity, and an increased risk of death (2–4). Unfortunately, there is no effective method to identify those AECOPD patients with poor prognosis. Oxidative stress plays an important role as an amplifying mechanism of inflammation in COPD (5). The imbalance between oxidants and antioxidants persists in stable COPD and is further elevated during exacerbations (6, 7). Previous studies showed that increased markers of oxidative stress such as malondialdehyde, advanced glycation end-products, 8-isoprostane, and 4-hydroxy-2-nonenal were associated with a poor prognosis in COPD (8, 9). However, another component of oxidative imbalance, antioxidants, have not been well explored in the prognosis of COPD.

Extracellular superoxide dismutase (ecSOD, SOD3) is the only extracellular scavenger of the superoxide radical and has been reported to be markedly higher in the lung (10). EcSOD plays a critical role in the regulation of lung-related oxidant inflammation (11). Loss of extracellular redox regulation promotes emphysema development (12). Genome-wide association studies show that polymorphisms in *SOD3* have been associated with declining lung function in COPD (13) and that smokers with elevated activity of serum ecSOD have a substantially lower risk for COPD (14). Thus, ecSOD could be a potential prognostic marker in COPD.

In COPD patients suffering from exacerbations, it was found that the antioxidant pathway was downregulated, and serum ecSOD activity was decreased (15, 16). Increased oxidative stress correlates positively with the severity of injury and poor prognosis in acute illness (17) and ecSOD activity negatively correlate with the severity of COPD exacerbations (18). We hypothesized that AECOPD patients with lower serum ecSOD activity has more severe oxidative stress during exacerbations, which leads to poorer outcomes. However, to our knowledge, there is no study that has investigated the prognostic role of serum ecSOD activity in AECOD patients. Therefore, we performed a prospective cohort study to ascertain the prognostic role of serum ecSOD activity for 1-year all-cause mortality in AECOPD.

## Methods

### Study Design and Patients

This prospective observational cohort study recruited consecutive hospitalized AECOPD patients in the Respiratory Department of the Third Affiliated Hospital of Guangzhou Medical University (Guangzhou, People's Republic of China) from January 01, 2016–January 10, 2020. Participants were diagnosed with COPD [with a documented diagnosis of post-bronchodilator forced expiratory volume in one second (FEV_1_)/forced vital capacity (FVC) ratio of <0.7] and AECOPD [defined as an increase in the severity of one or more respiratory symptoms (cough, sputum, wheezing, dyspnea, and/or chest tightness), requiring hospitalization] at admission, defined according to the Global Initiative for Chronic Obstructive Lung Disease (GOLD) criteria (19). Other diseases that worsen respiratory symptoms [pneumonia, pneumothorax, pleural effusion, pulmonary embolism, and dyspnea because of heart conditions (congestive heart failure (CHF), ischaemic heart disease (IHD), and arrhythmias)] were evaluated and ruled out in the differential diagnosis of COPD exacerbations. The primary diagnosis was AECOPD, and this diagnosis was made by respiratory physicians. This study did not exclude patients with coexisting asthma, obstructive sleep apnea-hypopnea syndrome, or bronchiectasis. Patients with active tuberculosis, pulmonary fibrosis, a pulmonary embolism, or a malignancy were excluded from the study. We also excluded patients who were unwilling to cooperate. Patients who were hospitalized for AECOPD multiple times during the study period were only included in the first hospitalization. Patients were treated by their attending physicians according to the GOLD guidelines. After discharge, patients were followed up every 3 months for 1 year by telephone. Follow-up started on the first day of discharge. This study was authorized by the Ethics Committee of Guangzhou's Third Affiliated Medical Hospital (No. 2016-004). All participants signed a written informed consent. This study was conducted in accordance with the Declaration of Helsinki. This article follows the STROBE reporting checklist (Supporting Information).

### Data Collection and Definitions

We extracted demographic (age, sex, smoking history, and body-mass index) and clinical (GOLD stage, number of exacerbations during the preceding year, a history of asthma, a history of obstructive sleep apnea-hypopnea syndrome, a history of bronchiectasis, a history of CHF, and a history of IHD) data from patients' electronic medical records. Prior to receiving any medications (except for the use of previously administered maintenance drugs), blood samples were collected from recruited hospitalized AECOPD patients, and a complete blood count, arterial blood gases (pH, partial pressure of O_2_ and partial pressure of CO_2_) and serum ecSOD activity were measured. The serum ecSOD activity was estimated using the pyrogallol autoxidation colorimetric kit (Ningbo Medicalsystem Biotechnology Co., Ltd., Ningbo, Zhejiang Province, China). All tests were performed by the hospital laboratory. A never-smoker was defined as smoking fewer than 100 cigarettes before the baseline. A current smoker was defined as smoking at baseline. A former smoker at baseline was defined as one who had smoked more than 100 cigarettes but had not smoked for at least 6 months (20). GOLD stage was classified into four groups based on FEV_1_ (GOLD 1: FEV_1_ ≥ 80% predicted; GOLD 2: 50% ≤ FEV_1_ <80% predicted; GOLD 3: 30% ≤ FEV_1_ <50% predicted; GOLD 4: FEV_1_ <30% predicted) (19).

### Statistical Analysis

The primary outcome was 1-year all-cause mortality. Parametric data were expressed as the mean ± standard deviation. Categorical variables were expressed as percentages. We compared groups using the independent samples *t*-test or chi-square test, as appropriate. We used the maximally selected log-rank statistics to obtain an optimal cut-off value of the correlation between ecSOD activity and 1-year all-cause mortality with the maxstat R package (21). The entire cohort of patients were divided into two groups based on the optimal cut-off value. We used the Kaplan–Meier estimator and log-rank test to determine the difference in 1-year all-cause mortality between the two groups. We performed a subgroup analysis to limit the influence of smoking and cardiovascular diseases (CHF and IHD) on ecSOD activity. Univariate and multivariate Cox regression were used for analyses of the relationship between ecSOD activity and 1-year all-cause mortality. We constructed three models to clarify the relationship between 1-year all-cause mortality and ecSOD activity. Model 1 only included ecSOD activity. Model 2 controlled for baseline covariates (age, sex, body mass index, smoking status, and pack-years). Model 3 was based on model 2 with the addition of comorbidities and clinical variables previously reported, related to COPD mortality [GOLD stage, exacerbations during the preceding year, and the partial pressure of CO_2_ (PaCO_2_)] (22–24). The proportional hazard assumption was examined by visual assessment of log-log survival curves against time, and there was no violation of this assumption. We used restricted cubic spline to flexibly model and visualize the relationship between serum ecSOD activity and the hazard ratio (HR) of 1-year all-cause mortality after adjustment for clinical variables and comorbidities. Participants lost to follow-up or with missing data would not be included in the analysis. The mortality of COPD among the exposed was unavailable, we were unable to estimate the sample size (25). All analyses were two-sided, and a *p*-value of <0.05 was considered statistically significant. Restricted cubic spline curves were performed using the RMS package in R (version 4.0.4), and other analyses were performed using SPSS software, version 16 (SPSS Inc., Chicago, IL, USA).

## Results

### Patient Characteristics

**Figure 1:**
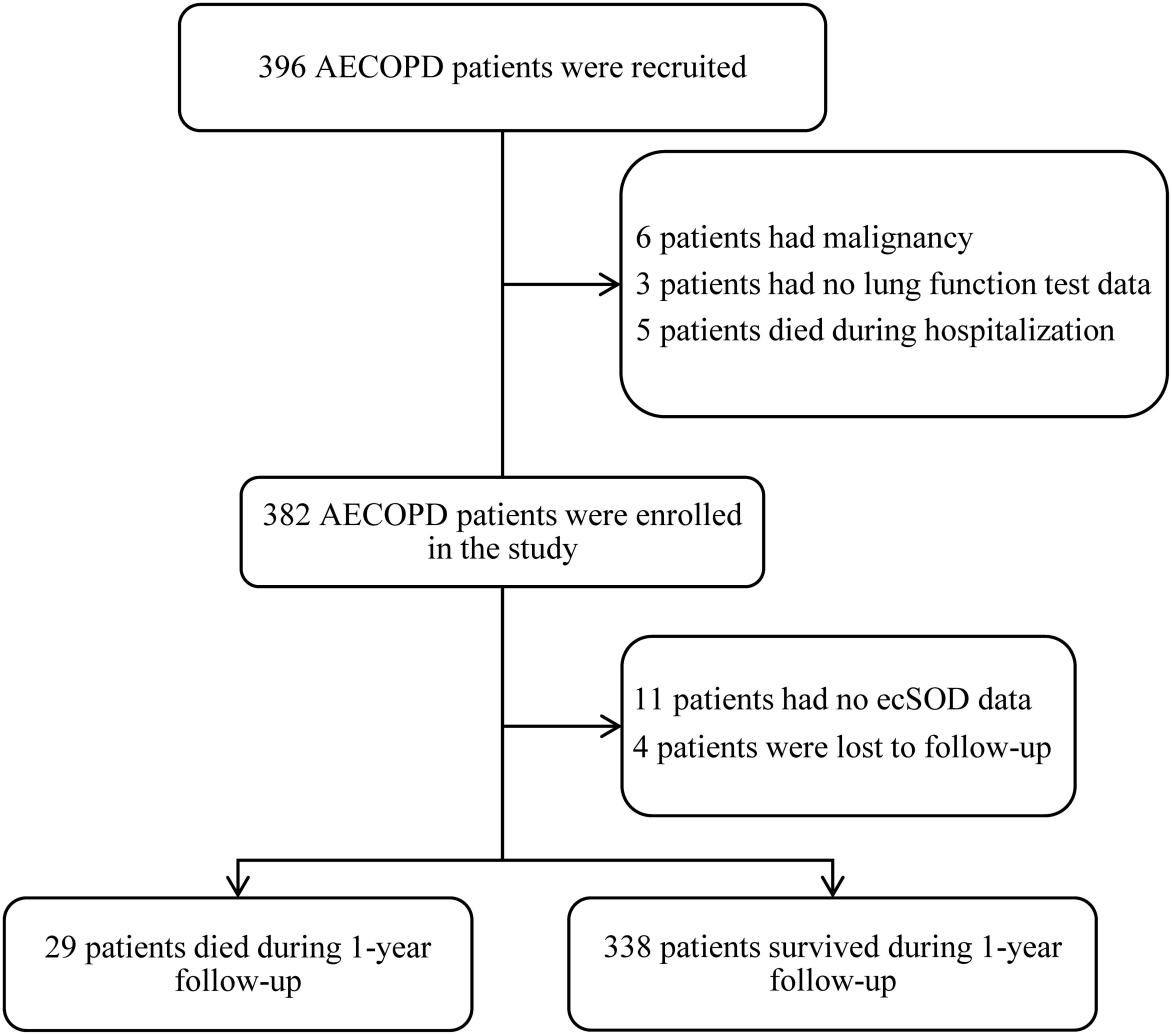
Flow chart of study patients.

A total of 396 consecutive patients were recruited and after screening for inclusion and exclusion criteria, 382 patients were enrolled, and ecSOD activity data for 11 of these patients were unavailable. Four patients were lost to follow-up after discharge, and 367 patients completed a 1-year follow-up and were included in the study analysis (Figure 1). The mean age of the patient was 74.78 ± 9.36 years and a total of 80.4% of patients were males. The demographic and baseline clinical features of patients between the survivor group and the non-survivor group are presented in Table 1. The non-survivor group was older (79.52 ± 8.39 vs. 74.38 ± 9.34 years old, *p* = 0.004) and had more COPD patients with GOLD stage four (24.1 vs. 16.6%) than did the survivor group. Compared with the survivor group, the non-survivor group also had more smokers (82.7 vs. 77.2%) and increased tobacco consumption (47.07 ± 41.67 vs. 33.83 ± 31.79 pack-years, *p* = 0.037).

**Table 1:** Characteristics of the patients according to 1-year all-cause mortality.

**Characteristic**	**All patients (367)**	**Non-survivors (*n* = 29)**	**Survivors (*n* = 338)**	***P*-value**
EcSOD	111.13 ± 19.56	94.37 ± 23.70	112.57 ± 18.50	<0.001
Age (yr.)	74.78 ± 9.36	79.52 ± 8.39	74.38 ± 9.34	0.004
Male sex (%)	80.4	72.4	81.1	0.260
Body-mass index (kg/m^2^)	21.42 ± 3.89	21.53 ± 3.53	20.99 ± 3.92	0.876
Smoking status (%)				0.515
Never smoking	22.3	17.2	22.8	
Former smoking	55.3	65.5	54.4	
Current smoking	22.3	17.2	22.8	
Tobacco consumption (pack-yr.)	34.88 ± 32.80	47.07 ± 41.67	33.83 ± 31.79	0.037
GOLD stage (%)				0.631
I	7.4	3.4	7.7	
II	36.5	37.9	36.4	
III	39.0	34.5	39.3	
IV	17.2	24.1	16.6	

*Plus–minus values are means ± standard deviation.*

*GOLD, Global Initiative for Chronic Obstructive Lung Disease; EcSOD, extracellular superoxide dismutase*.

### Survival and Risk Analyses of the Relationship Between ecSOD Activity and 1-Year All-Cause Mortality

**Figure 2:**
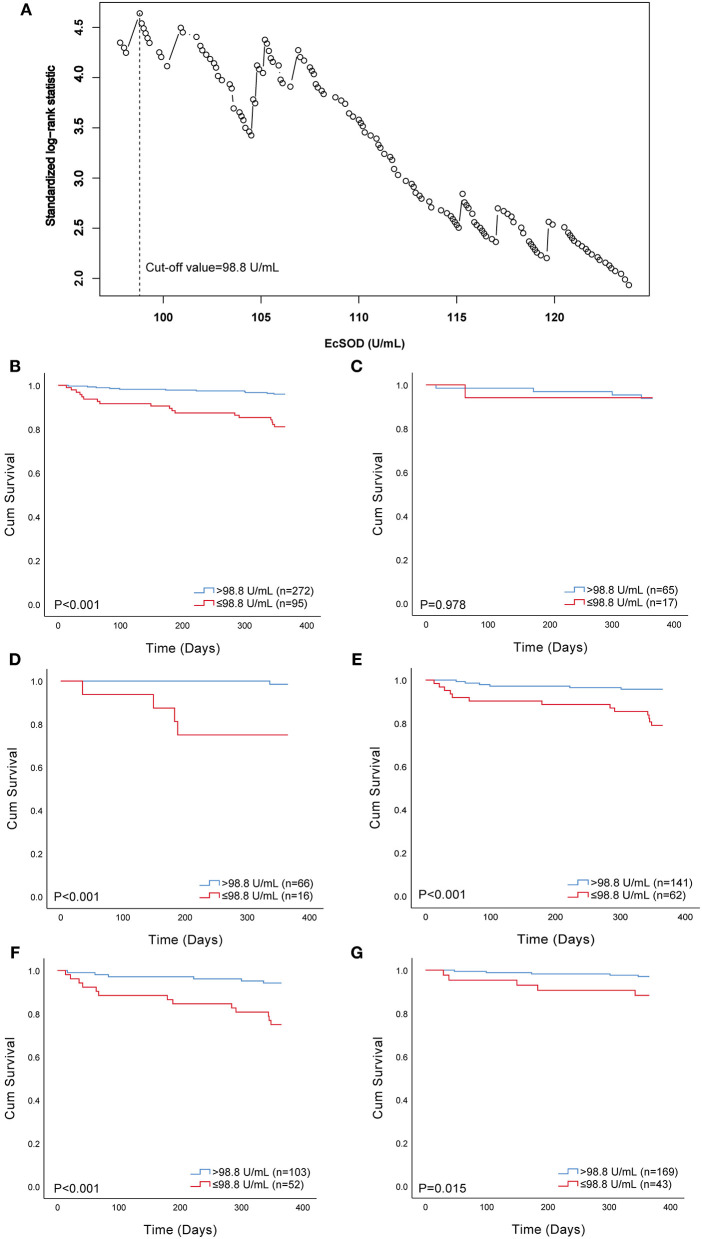
Selection of optimal cut-off value and Kaplan–Meier survival curves evaluating the time to death in days for patients. **(A)** standardized log-rank statistics for ecSOD optimal cut-off value; **(B)** survival curves of all patients; **(C)** survival curves of current smokers; **(D)** survival curves of never-smokers; **(E)** survival curves of former smokers; **(F)** survival curves of patients with CHF and/or IHD; **(G)** survival curves of patients without CHF and IHD. EcSOD, extracellular superoxide dismutase.

Twenty-nine individuals (7.90%) died in the entire cohort during follow-up. We first explored the association between 1-year all-cause mortality and ecSOD activity as a continuous variable with the use of Cox regression analysis. We found that ecSOD activity was negatively correlated with the risk of 1-year all-cause mortality [HR, 0.95; 95% confidence interval (CI): 0.93–0.97; *p* < 0.001). We separated the entire cohort into two groups according to the optimal cut-off value determined by maximally selected log-rank statistics to analyze differences in prognostic consequences (Figure 2A). The optimal cut-off value for ecSOD activity was 98.8 U/ml. The ecSOD activity ≤ 98.8 U/ml group had 95 patients.

The Kaplan–Meier estimator was used to reveal the difference in 1-year all-cause mortality between the ecSOD activity > 98.8 U/ml group and ecSOD activity ≤ 98.8 U/ml group (Figure 2B), and a significant difference was found between these two groups (*p* < 0.001). The survival rate in the ecSOD activity ≤ 98.8 U/ml group was lower than that in the ecSOD activity > 98.8 U/ml group (survival rate, 81.1 vs. 96.0, *p* < 0.001). In subgroup analysis, there were significant differences in survival curves between the two groups for both never smokers (survival rate, 75.0 vs. 98.5, *p* < 0.001, Figure 2D) and former smokers (survival rate, 79.0 vs. 95.7%, *p* < 0.001, Figure 2E). In the current smoking subgroup, the difference in survival curves between two groups was not significant (survival rate, 94.1 vs. 93.8%, *p* = 0.978, Figure 2C). In the CHF and/or IHD subgroup, the difference in survival curves between two groups was significant (survival rate, 75.0 vs. 94.2%, *p* = 0.978, Figure 2F). In the non-CHF and non-IHD subgroup, the difference in survival curves between two groups was significant (survival rate, 88.4 vs. 97.0%, *p* = 0.015, Figure 2G).

**Figure 3:**
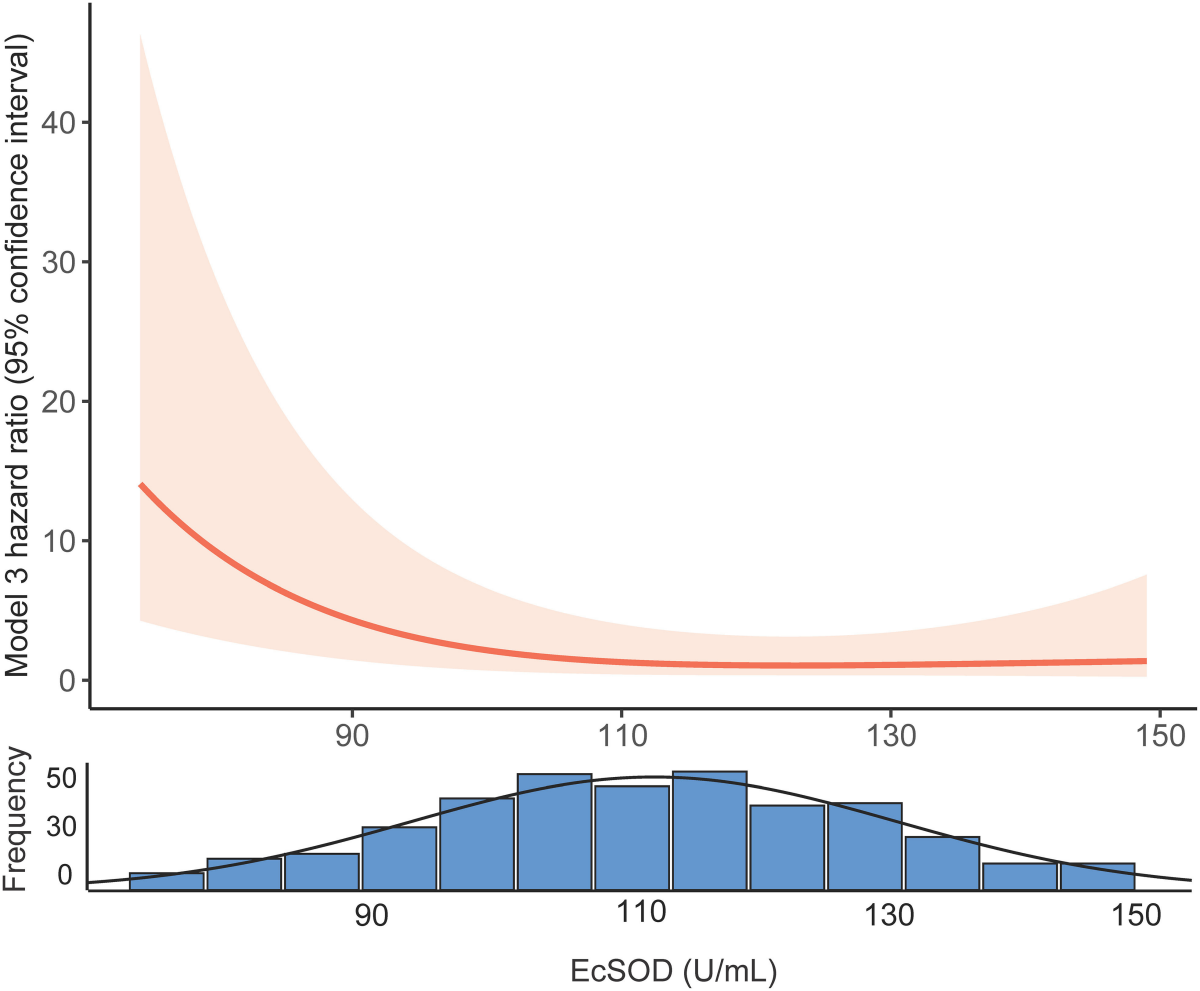
Restricted cubic spline curves of the association between ecSOD activity and 1-year all-cause mortality. EcSOD, extracellular superoxide dismutase.

We constructed three models to clarify the relationship between the ecSOD activity and 1-year all-cause mortality with the use of multivariate Cox proportional hazards analysis (Table 2). The unadjusted HR (model 1) for 1-year all-cause mortality in the ecSOD activity ≤ 98.8 U/ml group was 5.09 (95% CI: 2.41-10.78; *p* < 0.001). After adjusting for baseline characteristics (model 2), the HR was 4.11 (95% CI: 1.85-9.12; *p* = 0.001). After further adjustments for clinical variables and comorbidities (model 3), the relationship between the ecSOD activity ≤ 98.8 U/ml group and 1-year all-cause mortality remained significant (HR, 5.51; 95% CI: 2.35-12.95; *p* < 0.001). The restricted cubic spline curve (Figure 3), based on model 3 Cox proportional hazards, was used to visualize the association of 1-year all-cause mortality and ecSOD activity. The risk of 1-year all-cause mortality was relatively flat until ecSOD activity was <98.8 U/ml and then began to climb rapidly afterwards (*p* for non-linearity < 0.001).

**Table 2:** Multivariate Cox proportional hazards analysis of the association between ecSOD activity and 1-year all-cause mortality.

	**Model 1**		**Model 2**		**Model 3**	
**Variable**	**HR (95% CI)**	***P*-value**	**HR (95% CI)**	***P*-value**	**HR (95% CI)**	***P*-value**
EcSOD (per increase of 1 U/ml)	0.95 (0.93-0.97)	<0.001	-	-	-	-
EcSOD (≤ 98.8 vs. >98.8 U/ml)	5.09 (2.41-10.78)	<0.001	4.11 (1.85-9.12)	0.001	5.51 (2.35-12.95)	<0.001
Age (per increase of 10-year)			1.46 (0.86-2.48)	0.160	1.99 (1.05-3.77)	0.036
Sex (male vs. female)			0.37 (0.14-0.98)	0.046	0.32 (0.11-0.87)	0.026
Body-mass index (per increase of 1 point)			1.01 (0.92-1.11)	0.805	1.02 (0.92-1.12)	0.728
Smoking status						
Current smoking vs. never smoking			1.15 (0.28-4.77)	0.849	1.15 (0.64-2.04)	0.646
Former smoking vs. never smoking			1.35 (0.4-4.56)	0.629	1.08 (0.98-1.21)	0.131
Tobacco consumption (per increase of 10 pack-yr.)			1.09 (0.99-1.21)	0.067	1.82 (0.96-3.45)	0.066
GOLD stage (per increase to next stage)					0.81 (0.35-1.85)	0.611
Exacerbations during preceding year (yes vs. no)					2.75 (1.09-6.97)	0.033
PaCO_2_(≥50 vs. <50 mmHg)					2.81 (0.84-9.43)	0.095
Asthma (yes vs. no)					0.54 (0.23-1.28)	0.163
Bronchiectasis (yes vs. no)					1.56 (0.55-4.39)	0.402
OSAHS (yes vs. no)					0.99 (0.41-2.36)	0.978
CHF (yes vs. no)					1.78 (0.81-3.9)	0.154
IHD (yes vs. no)					1.15 (0.64-2.04)	0.646

*HR, hazard ratio; CI, confidence interval; ecSOD, extracellular superoxide dismutase; GOLD, Global Initiative for Chronic Obstructive Lung Disease; PaO_2_, partial pressure of O_2_; PaCO_2_, partial pressure of CO_2_; OSAHS, obstructive sleep apnea-hypopnea syndrome; CHF, congestive heart failure; IHD, ischaemic heart disease*.

### Association Between ecSOD Activity and Clinically Relevant Outcomes

Table 3 shows the clinical characteristics of the patients in both the activity ≤ 98.8 U/ml and the activity > 98.8 U/ml groups. There were significant differences between the two groups regarding age, a history of asthma, a history of CHF, a history of IHD, white blood cell count, percent neutrophils, red blood cell count and hemoglobin. EcSOD activity in COPD patients at different GOLD stages (I/II/III/IV: 110.13 ± 18.45/110.79 ± 19.77/109.42 ± 20.69/116.18 ± 16.20 U/ml, respectively, *p* = 0.145) or smoking status (current smoker/former smoker/never smoker: 113.81 ± 19.31/109.66 ± 20.15/112.09 ± 18.13 U/ml, respectively, *p* = 0.237) during exacerbations were not significantly different. For current smoker, the ecSOD activity in the non-survivor group and the survivor group were 111.80 ± 26.92 U/ml and 113.94 ± 18.95 U/ml (*p* = 0.812), respectively. For former smoker, the ecSOD activity in the non-survivor group and the survivor group were 92.49 ± 20.33 U/ml and 111.44 ± 19.33 U/ml (*p* <0.001), respectively. For never smoker, the ecSOD activity in the non-survivor group and the survivor group were 84.10 ± 28.65 U/ml and 113.91 ± 15.88 U/ml (*p* < 0.001), respectively.

**Table 3:** Characteristics of patients stratified by ecSOD activity.

**Characteristic**	**EcSOD ≤98.8 U/ml (*n* = 95)**	**EcSOD > 98.8 U/ml (*n* = 272)**	***P*-value**
Hospital stay (days)	8.28 ± 2.97	7.73 ± 2.65	0.088
Age (yr.)	79.22 ± 7.04	73.24 ± 9.59	<0.001
Male sex (%)	84.2	79.0	0.275
Body-mass index (kg/m^2^)	21.09 ± 4.07	21.54 ± 3.82	0.330
Smoking status (%)			0.076
Never smoking	16.8	24.3	
Former smoking	65.3	51.8	
Current smoking	17.9	23.9	
Tobacco consumption (pack-yr.)	40.35 ± 36.6	32.96 ± 31.21	0.059
GOLD stage (%)			0.175
I	6.3	7.7	
II	34.7	37.1	
III	47.4	36.0	
IV	11.6	19.1	
Exacerbations during preceding year (%)	47.4	44.5	0.627
The number of exacerbations during preceding year	1.16 ± 1.79	0.94 ± 1.48	0.238
Asthma (%)	3.2	9.9	0.038
Bronchiectasis (%)	38.9	29.8	0.100
OSAHS (%)	11.6	14.0	0.555
CHF (%)	32.6	21.7	0.033
IHD (%)	35.8	23.5	0.020
White blood cell count ( ×10^9^/L)	11.34 ± 5.4	9.24 ± 3.6	<0.001
Percent neutrophil (%)	79.14 ± 11.06	73.03 ± 12.22	<0.001
Percent eosinophils (%)	1.63 ± 2.85	2.19 ± 2.7	0.090
Red blood cell count ( ×10^12^/L)	4.23 ± 0.65	4.59 ± 0.69	<0.001
Hemoglobin (g/L)	122.65 ± 17.8	134.1 ± 18.37	<0.001
Platelet count ( ×10^9^/L)	256.8 ± 80.33	246.74 ± 73.7	0.264
pH	7.4 ± 0.04	7.39 ± 0.04	0.062
PaO_2_ (mmHg)	87.86 ± 26.75	91.17 ± 23.38	0.254
PaCO_2_ (mmHg)	43.06 ± 9.64	45 ± 10.51	0.115

*Plus–minus values are means ± standard deviation.*

*EcSOD, extracellular superoxide dismutase; GOLD, Global Initiative for Chronic Obstructive Lung Disease; OSAHS, obstructive sleep apnea-hypopnea syndrome; CHF, congestive heart failure; IHD, ischaemic heart disease; PaO_2_, partial pressure of O_2_; PaCO_2_, partial pressure of CO_2_*.

## Discussion

We investigated the association between serum ecSOD activity and 1-year all-cause mortality in our prospective cohort study of AECOPD. We found that lower serum ecSOD activity in AECOPD patients might be a significant predictor for their 1-year all-cause mortality. To our knowledge, this is the first study to highlight the relationship between ecSOD activity and 1-year all-cause mortality in AECOPD. Exacerbations of COPD were associated with airway and systemic oxidative stress-induced inflammation (26). EcSOD had been well-documented to be involved in the maintenance of extracellular oxidant-antioxidant balance and play an essential role in protecting the lung under inflammatory stress conditions (10). COPD was a systemic inflammatory disease (1), at least in part, serum ecSOD activity could reflect the oxidative imbalance in the lungs. Previous studies showed that the ecSOD activity in the serum (16, 27) or bronchoalveolar lavage fluid (28) of AECOPD patients was decreased compared with healthy control and stable COPD. Biologically, the changes in serum ecSOD activity were indicative of the oxidant/antioxidant imbalance (29). Therefore, we thought that decreased serum ecSOD activity in AECOPD patients would result in increased oxidative stress and a poor prognosis. Our study provided evidence that AECOPD patients with lower ecSOD activity had an increased risk of 1-year all-cause mortality.

The restricted cubic spline curve (Figure 3) visualized the relationship between serum ecSOD activity and the risk of 1-year all-cause mortality and demonstrated that the risk of 1-year all-cause mortality increases when ecSOD activity decrease below 98.8 U/ml. We also performed a subgroup analysis to clarify whether smoking had an influence on the prognostic effect and found that the difference in survival curves between the two groups (ecSOD activity ≤ 98.8 U/ml and ecSOD activity > 98.8 U/ml) remained significant for both former and never smokers, but not in the current smokers. The reason might be that ecSOD activity > 98.8 U/ml group had more patients with asthma, and the sample size of the current smoking subgroup was small. A previous study from Juul et al. (30) found that smokers who were *SOD3* R213G heterozygotes (mutation causes elevated serum ecSOD levels) had a decreased risk of hospitalization or mortality from COPD. Furthermore, Singh et al. (18) proposed that ecSOD activity negatively correlate with the severity of COPD exacerbations. Previous studies showed that CHF and IHD were associated with lower ecSOD activity (31, 32). Consistently with our study, the ecSOD activity ≤ 98.8 U/ml group had more patients with CHF and IDH. Furthermore, CHF and IHD were also important mortality risk factors for COPD patients (1). Therefore, we conducted a subgroup analysis to clarify the relationship between ecSOD activity and all-cause mortality in AECOPD patients without CHF and IHD. The relationship remained significant in the subgroup analysis (unadjusted HR, 5.10; 95% CI: 2.41–10.78; *p* < 0.001). These studies provided evidence that serum ecSOD activity had potential as a prognostic factor in AECOPD patients.

We also found that AECOPD patients with lower ecSOD activity were older, had less asthma, and had higher white blood cell counts, and percent neutrophils while their red blood cell counts, and hemoglobin levels were lower. Previous studies showed that smokers and stable COPD patients, both with low FEV_1_, were associated with decreased ecSOD activity (18, 33), but our study did not find these correlations. The main reason for the difference might be that COPD patients during exacerbations were in an extreme oxidation-antioxidant imbalance. Supplementing patients with antioxidants has been shown to be beneficial in preventing disease progression (10, 26). AECOPD patients with lower serum ecSOD activity might require additional antioxidant treatment. In another clinical trial, our research team is evaluating the effect of N-acetylcysteine on FEV_1_ in mild-to-moderate COPD to evaluate the role of antioxidants in COPD treatment (34). Nonetheless, a previous study suggested that antioxidants could increase the risk of tumorigenesis (35), indicating that antioxidant supplementation had two contrary sides. Notably, our data also demonstrated that as serum ecSOD activity increased to a certain level, the 1-year all-cause mortality did not change significantly (Figure 3). Therefore, finding the balance in oxidative stress could be of great significance for optimizing treatment.

This study had several limitations. First, this study was a single-center trial with relatively small died patients, but this was the largest prospective cohort that we know of that was used to explore the prognostic role of ecSOD activity in AECOPD. Regardless, a multicenter study is still needed to verify the association between ecSOD activity and 1-year all-cause mortality in AECOPD. Second, we did not exclude patients with coexisting asthma, bronchiectasis, or obstructive sleep apnea-hypopnea syndrome. These diseases are common comorbidities in COPD and their inclusion in the cohort made our study closer to a real-world study and more conducive to external validation. Moreover, we conducted multivariate analysis that included these comorbidities and found that it had minimal impact on the original model. Third, some factors that could affect ecSOD activity, such as patients receiving oxygen therapy at the outpatient or daily taking vitamins and supplements, were not restricted when collecting blood samples. It was very difficult to restrict patients from receiving oxygen therapy at the outpatient before collecting blood samples. In addition, a correlation analysis was performed to explore the relationship between PaO_2_ and ecSOD activity, and there was no correlation between them (Spearman rho = 0.002, *p* = 0.971). The influence of patients taking vitamins and other supplements daily on ecSOD activity was a long-term effect. We considered that the ecSOD activity was an antioxidative indicator that reflected the body's comprehensive antioxidant capacity. The effect of taking vitamins could be reflected by ecSOD activity. Our study aimed to explore the relationship between ecSOD activity during exacerbations and 1-year all-cause mortality, and lower SOD activity represented more severe oxidative stress in patients within exacerbations. Therefore, we did not exclude patients taking vitamins and other supplements. Fourth, we could not be certain whether serum ecSOD activity reflected those in the lungs. There is no direct evidence to show the relationship between serum ecSOD activity and lung ecSOD activity in COPD. However, asthma studies have shown that airways as well as blood monocytes and neutrophils had lower MnSOD activity in asthma patients than in control subjects (36).

## Data Availability Statement

With the permission of the corresponding authors, we can provide participant data without names and identifiers after publication. A proposal with a detailed description of study objectives and a statistical analysis plan will be needed for evaluation of the reasonability of requests. The corresponding authors have the right to decide whether to share the data or not based on the research objectives and plan provided.

## Ethics Statement

The studies involving human participants were reviewed and approved by the Ethics Committee of Guangzhou's Third Affiliated Medical Hospital. The patients/participants provided their written informed consent to participate in this study.

## Author Contributions

GH and PR conceived the idea for this report. HL wrote the first draft of the article. HL, WH, GH, LW, YZ, and PR contributed to the final version. HL, ZZ, SG, JC, ZW, MG, RS, WC, and YL collected data for the study. HL, FW, HT, and GH performed the statistical analyses. All authors contributed to the article and approved the submitted version.

## Funding

This study was supported by the National Natural Science Foundation of China (81670042 and 82000045), the Natural Science Foundation of Guangdong Province (2017A030313887), the Guangzhou Science and Technology Project (201707010232), and the Local Innovative and Research Teams Project of Guangdong Pearl River Talents Program (2017BT01S155).

## Conflict of Interest

The authors declare that the research was conducted in the absence of any commercial or financial relationships that could be construed as a potential conflict of interest.

## Publisher's Note

All claims expressed in this article are solely those of the authors and do not necessarily represent those of their affiliated organizations, or those of the publisher, the editors and the reviewers. Any product that may be evaluated in this article, or claim that may be made by its manufacturer, is not guaranteed or endorsed by the publisher.

## References

[1] Singh D Agusti A Anzueto A Barnes PJ Bourbeau J Celli BR. Global strategy for the diagnosis, management, and prevention of chronic obstructive lung disease: the gold science committee report 2019. Eur Respir J. (2019) 53:1900164. 10.1183/13993003.00164-2019 30846476

[2] Seemungal TA Donaldson GC Paul EA Bestall JC Jeffries DJ Wedzicha JA. Effect of exacerbation on quality of life in patients with chronic obstructive pulmonary disease. Am J Respir Crit Care Med. (1998) 157:1418-22. 10.1164/ajrccm.157.5.9709032 9603117

[3] Donaldson GC Seemungal TA Bhowmik A Wedzicha JA. Relationship between exacerbation frequency and lung function decline in chronic obstructive pulmonary disease. Thorax. (2002) 57:847-52. 10.1136/thorax.57.10.847 PMC1746193 12324669

[4] Soler-Cataluna JJ Martinez-Garcia MA Roman SP Salcedo E Navarro M Ochando R. Severe acute exacerbations and mortality in patients with chronic obstructive pulmonary disease. Thorax. (2005) 60:925-31. 10.1136/thx.2005.040527 PMC1747235 16055622

[5] Domej W Oettl K Renner W. Oxidative stress and free radicals in copd–implications and relevance for treatment. Int J Chron Obstruct Pulmon Dis. (2014) 9:1207-24. 10.2147/COPD.S51226 PMC4207545 25378921

[6] Kirkham PA Barnes PJ. Oxidative stress in copd. Chest. (2013) 144:266-73. 10.1378/chest.12-2664 23880677

[7] Zinellu E Zinellu A Fois AG Carru C Pirina P. Circulating biomarkers of oxidative stress in chronic obstructive pulmonary disease: a systematic review. Respir Res. (2016) 17:150. 10.1186/s12931-016-0471-z 27842552 PMC5109807

[8] Gopal P Reynaert NL Scheijen JL Engelen L Schalkwijk CG Franssen FM. Plasma advanced glycation end-products and skin autofluorescence are increased in copd. Eur Respir J. (2014) 43:430-8. 10.1183/09031936.00135312 23645408

[9] McGuinness AJ Sapey E. Oxidative stress in copd: sources, markers, and potential mechanisms. J Clin Med. (2017) 6:21. 10.3390/jcm6020021 28212273 PMC5332925

[10] Kinnula VL Crapo JD. Superoxide dismutases in the lung and human lung diseases. Am J Respir Crit Care Med. (2003) 167:1600-19. 10.1164/rccm.200212-1479SO 12796054

[11] Arcaroli JJ Hokanson JE Abraham E Geraci M Murphy JR Bowler RP. Extracellular superoxide dismutase haplotypes are associated with acute lung injury and mortality. Am J Respir Crit Care Med. (2009) 179:105-12. 10.1164/rccm.200710-1566OC PMC2633057 18948423

[12] Yao H Arunachalam G Hwang JW Chung S Sundar IK Kinnula VL. Extracellular superoxide dismutase protects against pulmonary emphysema by attenuating oxidative fragmentation of ecm. Proc Natl Acad Sci USA. (2010) 107:15571-6. 10.1073/pnas.1007625107 PMC2932580 20713693

[13] Dahl M Bowler RP Juul K Crapo JD Levy S Nordestgaard BG. Superoxide dismutase 3 polymorphism associated with reduced lung function in two large populations. Am J Respir Crit Care Med. (2008) 178:906-12. 10.1164/rccm.200804-549OC PMC2577726 18703790

[14] Young RP Hopkins R Black PN Eddy C Wu L Gamble GD. Functional variants of antioxidant genes in smokers with copd and in those with normal lung function. Thorax. (2006) 61:394-9. 10.1136/thx.2005.048512 PMC2111196 16467073

[15] Lin JL Thomas PS. Current perspectives of oxidative stress and its measurement in chronic obstructive pulmonary disease. Copd. (2010) 7:291-306. 10.3109/15412555.2010.496818 20673039

[16] Antus B Paska C Simon B Barta I. Monitoring antioxidant enzyme activity during exacerbations of chronic obstructive pulmonary disease. Copd. (2018) 15:496-502. 10.1080/15412555.2018.1535581 30475645

[17] Bar-Or D Bar-Or R Rael LT Brody EN. Oxidative stress in severe acute illness. Redox Biol. (2015) 4:340-5. 10.1016/j.redox.2015.01.006 PMC4326179 25644686

[18] Singh S Verma SK Kumar S Ahmad MK Nischal A Singh SK. Evaluation of oxidative stress and antioxidant status in chronic obstructive pulmonary disease. Scand J Immunol. (2017) 85:130-7. 10.1111/sji.12498 28256060

[19] Vestbo J Hurd SS Agusti AG Jones PW Vogelmeier C Anzueto A. Global strategy for the diagnosis, management, and prevention of chronic obstructive pulmonary disease: gold executive summary. Am J Respir Crit Care Med. (2013) 187:347-65. 10.1164/rccm.201204-0596PP 22878278

[20] Zhou Y Hu G Wang D Wang S Wang Y Liu Z. Community based integrated intervention for prevention and management of chronic obstructive pulmonary disease (copd) in guangdong, china: cluster randomised controlled trial. BMJ. (2010) 341:c6387. 10.1136/bmj.c6387 21123342 PMC2995286

[21] Hothorn T Lausen B. On the exact distribution of maximally selected rank statistics. Comput Stat Data Analy. (2003) 43:121-37. 10.1016/S0167-9473(02)00225-6

[22] Moll M Qiao D Regan EA Hunninghake GM Make BJ Tal-Singer R. Machine learning and prediction of all-cause mortality in copd. Chest. (2020) 158:952-64. 10.1016/j.chest.2020.02.079 PMC7478228 32353417

[23] Gudmundsson G Ulrik CS Gislason T Lindberg E Brøndum E Bakke P. Long-term survival in patients hospitalized for chronic obstructive pulmonary disease: a prospective observational study in the nordic countries. Int J Chron Obstruct Pulmon Dis. (2012) 7:571-6. 10.2147/COPD.S34466 PMC3459657 23055707

[24] Bellou V Belbasis L Konstantinidis AK Tzoulaki I Evangelou E. Prognostic models for outcome prediction in patients with chronic obstructive pulmonary disease: systematic review and critical appraisal. BMJ. (2019) 367:l5358. 10.1136/bmj.l5358 31585960 PMC6776831

[25] Schlesselman JJ. Sample size requirements in cohort and case-control studies of disease. Am J Epidemiol. (1974) 99:381-4. 10.1093/oxfordjournals.aje.a121625 4601871

[26] Dal Negro RW Visconti M Turco P. Efficacy of erdosteine 900 versus 600 mg/day in reducing oxidative stress in patients with copd exacerbations: results of a double blind, placebo-controlled trial. Pulm Pharmacol Ther. (2015) 33:47-51. 10.1016/j.pupt.2015.06.004 26116425

[27] Liu LY Zeng M Xie CM Gao JH Yan YS Lu GF. Oxidative stress status in patients with chronic obstructive pulmonary disease and its relation to glucocorticoid receptor levels. Nan Fang Yi Ke Da Xue Xue Bao. (2008) 28:992-6. 10.3321/j.issn:1673-4254.2008.06.037 18583246

[28] Zhao X Wu Y. Correlations of silent information regulator of transcription 1 (sirt1) expression, inflammatory factors, and oxidative stress with pulmonary function in patients with acute exacerbation of chronic obstructive pulmonary disease (aecopd). Med Sci Monit. (2021) 27:e929046. 10.12659/MSM.929046 33762567 PMC8008970

[29] MacNee W. Pulmonary and systemic oxidant/antioxidant imbalance in chronic obstructive pulmonary disease. Proc Am Thorac Soc. (2005) 2:50-60. 10.1513/pats.200411-056SF 16113469

[30] Juul K Tybjaerg-Hansen A Marklund S Lange P Nordestgaard BG. Genetically increased antioxidative protection and decreased chronic obstructive pulmonary disease. Am J Respir Crit Care Med. (2006) 173:858-64. 10.1164/rccm.200509-1387OC 16399992

[31] Castro PF Miranda R Verdejo HE Greig D Gabrielli LA Alcaino H. Pleiotropic effects of atorvastatin in heart failure: role in oxidative stress, inflammation, endothelial function, and exercise capacity. J Heart Lung Transplant. (2008) 27:435-41. 10.1016/j.healun.2008.01.012 18374881

[32] Landmesser U Merten R Spiekermann S Buttner K Drexler H Hornig B. Vascular extracellular superoxide dismutase activity in patients with coronary artery disease: relation to endothelium-dependent vasodilation. Circulation. (2000) 101:2264-70. 10.1161/01.CIR.101.19.2264 10811593

[33] Al-Azzawi MA Ghoneim AH Elmadbouh I. Evaluation of vitamin d, vitamin d binding protein gene polymorphism with oxidant - antioxidant profiles in chronic obstructive pulmonary disease. J Med Biochem. (2017) 36:331-40. 10.1515/jomb-2017-0012 PMC6294086 30581330

[34] Tian H Zhou Y Tang L Wu F Deng Z Lin B. High-dose n-acetylcysteine for long-term, regular treatment of early-stage chronic obstructive pulmonary disease (gold i-ii): study protocol for a multicenter, double-blinded, parallel-group, randomized controlled trial in china. Trials. (2020) 21:780. 10.1186/s13063-020-04701-8 32917271 PMC7488567

[35] Bjelakovic G Nikolova D Gluud LL Simonetti RG Gluud C. Mortality in randomized trials of antioxidant supplements for primary and secondary prevention: systematic review and meta-analysis. JAMA. (2007) 297:842-57. 10.1001/jama.297.8.842 17327526

[36] Vachier I Damon M Le Doucen C de Paulet AC Chanez P Michel FB. Increased oxygen species generation in blood monocytes of asthmatic patients. Am Rev Respir Dis. (1992) 146:1161-6. 10.1164/ajrccm/146.5_Pt_1.1161 1443865

